# Clinical relevance of serum antibodies to extracellular *N*-methyl-d-aspartate receptor epitopes

**DOI:** 10.1136/jnnp-2014-308736

**Published:** 2014-09-22

**Authors:** Michael S Zandi, Ross W Paterson, Mark A Ellul, Leslie Jacobson, Adam Al-Diwani, Joanne L Jones, Amanda L Cox, Belinda Lennox, Maria Stamelou, Kailash P Bhatia, Jonathan M Schott, Alasdair J Coles, Dimitri M Kullmann, Angela Vincent

**Affiliations:** 1 Department of Clinical Neurosciences, Addenbrooke’s Hospital, Cambridge, UK; 2 National Hospital for Neurology and Neurosurgery, UCL Institute of Neurology, London, UK; 3 Neurosciences Group, Nuffield Department of Clinical Neurosciences, John Radcliffe Hospital, Oxford, UK; 4 Department of Psychiatry, Warneford Hospital, Oxford, UK

## Abstract

**Objective:**

There are now a large number of requests for *N*-methyl-d-aspartate receptor autoantibody (NMDAR-Ab) tests, and it is important to assess the clinical relevance of all results, particularly when they are reported as ‘Low Positive’.

**Methods:**

The clinical data of 56 patients found Positive or Low Positive by the Oxford live cell-based assay were reviewed. An autoimmune basis for the condition was assigned as ‘Definite’, ‘Possible’ or ‘Unlikely’. The number of core features (encephalopathy, psychiatric, cognitive, epileptic, extrapyramidal and inflammatory cerebrospinal fluid (CSF)) was tabulated.

**Results:**

Twenty-five (44.6%) patients had a Definite NMDAR-Ab encephalitis (eight ovarian teratomas, one Hodgkin’s lymphoma), 18 (32.1%) a Possible NMDAR-Ab encephalitis and 13 (23.2%) an Unlikely autoimmune syndrome. Serum NMDAR-Ab levels were higher in patients with tumours. Positive NMDAR-Abs were found not only in patients with three or more core features and a Definite syndrome, but also in five patients classified as Possible. Conversely, Low Positive NMDAR-Abs were present in 7 Definite cases as well as in 13 Possible cases. Unlikely patients had mainly Low Positive antibodies and fewer core features. CSF NMDAR-Abs, only available in 11 pairs and at varying time points, broadly related to serum levels and were Positive in 3/3 patients with tumours but in only 2/5 Definite patients, and none of the Possible or Unlikely cases.

**Interpretation:**

Using live cell-based assays, Positive and Low Positive antibodies can be of clinical significance. The number of core clinical features should help to select those patients in whom an immunotherapy intervention might be considered, irrespective of the antibody level.

## Introduction

*N*-methyl-d-aspartate receptor autoantibodies (NMDAR-Abs), detected by binding to HEK293 cells expressing NMDAR, were first described in 2007 in young women who had presented with an encephalitis characterised by a psychiatric prodrome, seizures, rhythmic movement disorder, dysautonomia, coma and the presence of an ovarian teratoma.^1^ With increased recognition, the phenotype has broadened to include men, children and older individuals with subacute encephalopathies, sometimes with restricted phenotypes and including many patients without tumours.^2^ ^3^ The patients respond to tumour removal, if relevant, and immunotherapies including corticosteroids and intravenous immunoglobulins or plasmapheresis.^2^ In many patients, second-line agents such as cyclophosphamide or rituximab may be necessary to achieve best outcomes.^3^

Most NMDAR-Abs are directed towards the NR1 subunit.^4^ NMDAR-Abs are measured by cell-based assays (CBA) that detect serum or cerebrospinal fluid (CSF) IgG binding to the NR1 subunit expressed in HEK293 cells. These are fixed and permeabilised before sample application in the laboratory of Professor Josep Dalmau,^4^ and the most commonly employed commercial assay also uses fixed permeabilised cells. In contrast, the Oxford laboratory uses unfixed live HEK293 cells expressing NMDAR NR1 and NR2B subunits (found previously to obtain better surface expression of NR1^2^) with the aim of detecting only antibodies that bind to extracellular epitopes as these are the antibodies that are likely to be pathogenic.^5^–^7^ The NMDAR-Abs were originally demonstrated to be 100% specific for the syndrome as described^2^ ^4^ but have now also been reported in a proportion of patients with partial phenotypes including idiopathic localisation-related epilepsy and first episode psychosis.^2^ ^8^–^11^ Moreover, with increasing and earlier serum referrals, there have been reports of positive results in patients who are subsequently given an alternative diagnosis. For instance, Low Positive levels of NMDAR-Ab were reported in two patients who were diagnosed later with prion disease.^12^ While it is certainly possible that there is an additional autoimmune mechanism in some highly destructive neurological diseases, such low levels of NMDAR-Abs may be epiphenomena. On the other hand, the high levels of NMDAR-Abs recently found in children with relapses after established herpes virus encephalitis, a few of whom have shown immunotherapy responses,^13^ ^14^ are likely to be relevant, suggesting that even antibodies appearing following a neuronal damage can be pathogenic.

To assess afresh the clinical relevance of NMDAR-Abs at different levels, we identified all patients, from two UK centres, who were reported Positive or Low Positive for NMDAR-Abs since testing began in Oxford in 2008. We assessed the clinical details and treatment responses, and established the likelihood that they had an autoimmune immunotherapy-responsive disease.

## Methods

### Patients and clinical data

A total of 1039 patients (714 from Addenbrooke’s Hospital, Cambridge and 325 from the National Hospital for Neurology and Neurosurgery, Queen Square, London) had first sera referred to Oxford for NMDAR and other antibodies during 2008–2012. The records of patients with initial Positive or Low Positive NMDAR-Abs were reviewed retrospectively. The following variables were prospectively chosen as items of interest prior to review: age at presentation, neurological features and final clinical diagnosis, time from symptom onset to antibody testing in months, presence of tumour, CSF analysis, EEG and MRI findings, response to symptomatic treatments or immunotherapies and length of follow-up (time of the antibody test to the last documented clinical review). Some of the cases have been reported previously as part of other series and case reports^2^ ^15^ ^16^ (see Supplementary table 1). We excluded the 13 patients with NMDAR-Abs referred from psychiatric units in Cambridge for a specific study of antibodies in psychosis (J Deakin, M Zandi, B Lennox, A Vincent in preparation). Whether or not a tumour search was undertaken is indicated in the online supplementary table; the nature of the imaging varied between patients, including ovarian ultrasound, CT imaging and PET or a combination in some cases. Patients are listed in table 1 as paraneoplastic irrespective of the tumour type.

### Serology

NMDAR-Abs were measured by indirect cell surface immunocytochemistry of transfected cells^2^ as used for routine diagnostic assays by the Oxford Neuroimmunology service since 2008. Briefly, live HEK293 cells are transfected with cDNAs of NR1 and NR2B subunits and incubated in human serum (1:20 initial dilution) or CSF (1:1 initial dilution) before fixation, followed by Alexa-Fluor labelled antihuman IgG. Antibodies binding to the cell surface of NMDAR-HEK293 cells are identified by fluorescence microscopy. A visual scoring system (0–4) is used as previously^2^ and all results are confirmed by a second independent observer; positive samples are then repeated in a separate assay where they are also checked for specificity by lack of binding to HEK293 cells expressing an alternative antigen. No clinical data are available to the laboratory at the time. As with other assays, scores of 0 are reported as negative and scores of 2–4 were reported as Positive. Scores of 1 were initially reported as Low Positive, but after 2011 only scores of 1.5 were reported as Low Positive.

### Autoimmune likelihood of patients’ disease

To review the clinical relevance, each neurologist who cared for one or more of the patients (often one of the authors) was asked to provide a diagnosis. In most cases this was a clinical diagnosis, aided by the emerging reports of NMDAR encephalitis in the literature and discussions with one or more of the authors during the patient management. The diagnosis and treatment had been completed prior to this service review, but the authors made judgements regarding cases in which the NMDAR-Ab result came to light after a patient’s illness or death, or if the patient was not seen by a neurologist. Subsequently, the clinicians were asked to consider the response to immunotherapies to help categorise the patients as Definite NMDAR-encephalitis, a Possible NMDAR-antibody-mediated disease or an Unlikely NMDAR-Ab-mediated or autoimmune disease.^5^ ^17^

To determine how the antibody levels and these categories related to the core clinical features of NMDAR-Ab encephalitis (encephalopathy, psychiatric symptoms, cognitive symptoms, seizures, extrapyramidal movement disorder), the number of features including an inflammatory CSF (cellular, raised protein or oligoclonal bands if tested) were tabulated. We did not include slow waves on EEG as this feature would be captured by the presence of encephalopathy.

### Statistical analyses

The cell-based assay NMDAR scores for each group were compared with ANOVA, with post hoc comparisons.

## Results

**Figure 1:**
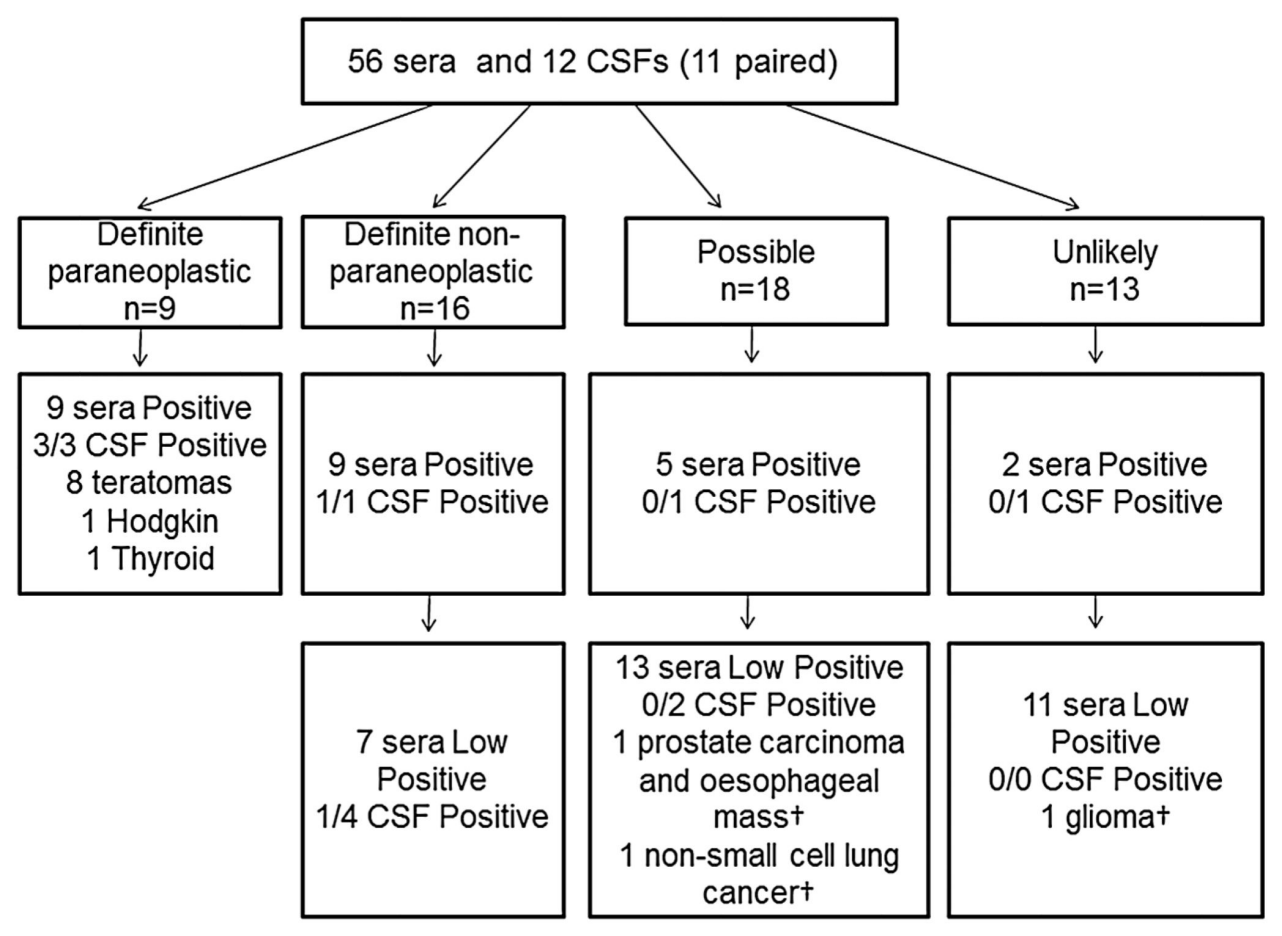
Schematic representation of the breakdown of patients into four clinical categories. The patients are divided into those with Positive and Low Positive initial NMDAR cell-based assays score. Cerebrospinal fluid (CSF) *N*-methyl-d-aspartate receptor autoantibody (NMDAR-Ab) and tumour associations (with mortality, †) are represented.

Of the 1039 patients tested, 56 (5.4%) were Positive or Low Positive for NMDAR-Abs in serum and of these, 9 were classified as paraneoplastic Definite NMDAR-Ab encephalitis (16%), 16 (29%) as non-paraneoplastic Definite NMDAR-Ab encephalitis, 18 (32%) as Possible NMDAR-Ab encephalitis and 13 (23%) as Unlikely autoimmune encephalitis (figure 1).

Only two cases (#28, #32) were redefined by the authors (MSZ and AJLC) on the basis that one previously assigned Definite case (#28) did not demonstrate improvement (before leaving the country), and one previously assigned Unlikely case (#32) did have clinical and paraclinical features that the authors considered made NMDAR-Ab encephalitis possible.

**Figure 2:**
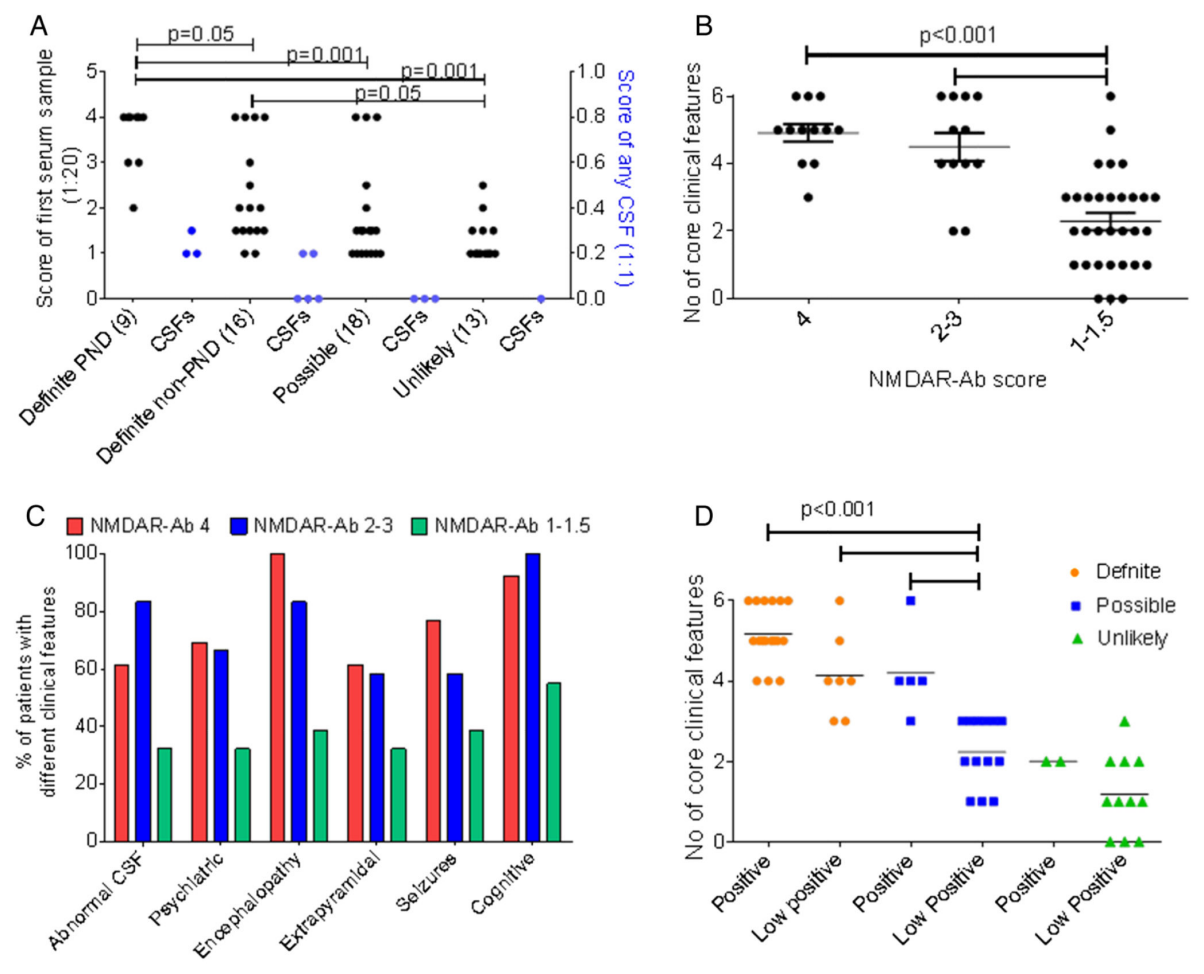
Distribution of cell-based serum and CSF NMDAR-Ab scores in each clinical category (A). The CBA score of the first serum sample at 1:20 titration and CSF at 1:1 (some tested subsequently, online supplementary table S1) score plotted against the clinical allocations. Definite paraneoplastic cases had significantly higher initial CBA scores than each of the other case definitions. (B) The number of core features plotted against the CBA scores: 4, 2–3 and 1–1.5. The numbers of core features were lower in the Low Positive group. (C) The percentage of patients with each core feature was compared for the different CBA scores. There were no clear differences related to antibody level. (D) The number of core clinical features was compared across clinical definitions and the binary allocation of Low Positive or Positive initial NMDAR CBA scores. Although the number of features was lower in the Possible Low Positive patients than in the Definite or Positive Possible patients, half of them had three core features. CBA, cell-based assay; CSF, cerebrospinal fluid; NMDAR-Ab, *N*-methyl-d-aspartate receptor autoantibody; PND, paraneoplastic neurological disorder.

The initial (diagnostic) NMDAR-Ab scores for each of these categories are shown in figure 2A. Initially, values of 1 were reported to clinicians as Low Positive and values from 2 to 4 were reported as Positive. Subsequently (see below), values of 1 were reported as negative and 1.5 as Low Positive. Details of the individual cases are identified by # in the online supplementary table S1 and the clinical features, paraclinical investigations, treatment status and outcomes in the Positive and Low Positive groups are summarised in table 1.

### Definite paraneoplastic

Nine patients had tumours; eight females (ages 17–32) with ovarian teratomas (one also with papillary thyroid cancer) and one male with Hodgkin’s lymphoma. Excluding one case with censored details, all had four to six of the core clinical features (encephalopathy, psychiatric symptoms, cognitive symptoms, seizures, extrapyramidal movement disorder and inflammatory CSF), and eight of nine had slowing on EEG; these patients responded well to immunotherapies. Patient #9 was a man with Hodgkin’s lymphoma and paraneoplastic limbic encephalitis (Ophelia syndrome^15^) with MRI evidence of medial temporal lobe involvement. He responded well initially but died subsequently of his Hodgkin’s lymphoma.

These patients had Positive NMDAR-Abs at diagnosis with overall higher scores than those in each of the other three classifications, and all three paired CSFs were Positive (figure 2A; table 1 and online supplementary table S1).

### Definite non-paraneoplastic

Sixteen patients (10 females, 6 males) were designated Definite on the basis of having a subacute or acute disorder with three (n=2) or more (n=14) of the six core clinical features, including 12 abnormal CSFs (10 cellular, 8 with oligoclonal bands), absence of a clear alternative diagnosis, and a response to immunotherapies in 15/16 (see table 1 and online supplementary table S1). One patient (#13) with classical NMDAR encephalitis and Positive NMDAR-Abs (previously reported in Davies *et al*^14^) was assigned as ‘Definite’ despite no response to therapy within the 6 months before he died. Another (#21) presented with an encephalitic illness with some recovery after immunotherapy, but then suffered progressive cognitive decline until death. A postmortem demonstrated evidence of Alzheimer’s disease and encephalitis.

At first testing, 9 of the 16 Definite non-paraneoplastic patients had Positive serum NMDAR-Abs (scores 2–4 at 1:20 dilution) and 1 paired CSF was Positive (scores 1 at 1:1 dilution). However, the remaining seven cases had Low Positive serum NMDAR-Ab scores (1 or 1.5) at presentation, and only two of the five paired CSFs was Positive this patient (#19) was tested when serum NMDAR-Ab had risen to Positive during the course of a protracted illness.

### Possible

Eighteen patients, seven women, median age 55 (range 22–80), were classified as Possible. Two with Low Positive NMDAR-Abs had tumours identified (#26, #27). Overall, 11 of 18 patients were encephalopathic and 9 of 15 tested had an inflammatory CSF (8 cellular, 1 with OCB). Although three or more core features were present in 11 patients, the remaining 7 patients had only one or two of these features. Six of the patients had received immunotherapies and only one patient (#34), with isolated psychosis, improved, although this young woman also received conventional antipsychotic medication. One notable case (#28) was a patient with severe encephalitis and venous sinus thrombosis, unresponsive to immunotherapy, initially thought to have neuro-Behçet’s disease. The Positive antibody result led the treating neurologist to make a diagnosis of Possible NMDAR-Ab encephalitis, and as a consequence change therapy from tumour necrosis factor (TNF) blockade to B-cell depletion therapy with rituximab. There was no convincing therapeutic effect, although the follow-up was short (the patient left the UK).

Five Possible cases had Positive NMDAR-Abs at first testing, and of the 13 (72.2%) with Low Positive serum NMDAR-Abs, four developed Positive values subsequently. The three CSFs tested during the course of the disease were negative (figure 2A).

### Unlikely cases

These 13 cases, median age of 52 (range 17–77), including 4 females, had an alternative eventual diagnosis or an antibody-mediated CNS syndrome was felt to be unlikely. One case had a primary brain malignancy (posterior fossa glioma, not biopsied before death), but there were no systemic malignancies in this group. This group included two cases of atypical motor neuron disease. In addition, there was one patient with leukodystrophy, a case of CNS vasculitis, a possible flare of maple syrup urine disease and one patient with an idiopathic pure cerebellar syndrome (see supplementary table S1). Only one case had three or more core clinical features and CSF was abnormal in only 2/12 cases (see supplementary table S1). Two patients (#50 and #55) had an immunotherapy trial but did not respond.

Two others were receiving immunotherapy for CNS vasculitis and systemic lupus erythematosus—not with the intention to treat an autoimmune encephalopathy—and similarly did not respond. One case with complex partial seizures and cognitive dysfunction, felt clinically to have possible early Alzheimer’s disease, had Positive NMDAR-Abs at onset rising to a score of 3 in a subsequent sample, but immunotherapies were not tried.

All but two patients (84.6%) had Low Positive NMDAR-Abs, and CSF was negative for NMDAR-Abs in the one case tested.

### Relationship of clinical features to NMDAR-Ab levels

The number of core clinical features, including inflammatory CSF, was significantly lower in patients with Low Positive NMDAR-Ab levels compared to those with Positive levels (either score 4 or scores 2–3, figure 2B; p<0.001) across the range of core features (figure 2C). Nevertheless, three or more core clinical features were found not only in all of those with Definite NMDAR-Ab encephalitis, including the 7 with Low Positive antibodies, but also in 11 of the Possible patients including 6 who were Low Positive (figure 2D). Overall, serum antibodies were higher than CSF in paired samples, and in some patients serum levels rose from Low Positive to Positive during the course of the disease (online supplementary table S1).

### Effect of omitting scores of 1

Over the first 4 years, we became aware that scores of 1 were relatively common and could be associated with unlikely autoimmune diagnoses. As from late 2011, we reported scores of 1 as negative and 1.5 as Low Positive. If this cut-off had been applied to all patients, it would have reduced the total number to 39, with 23 Definite, 11 Possible and only 5 Unlikely.

## Discussion

NMDAR-Abs are associated with a well-described disease that presents with psychosis, seizures, encephalopathy, cognitive impairment and movement disorders, often with CSF pleocytosis or oligoclonal bands. Although ‘anti-NMDAR encephalitis’ has been characterised in great detail,^2^ ^4^ ^6^ the range of clinical phenotypes is widening.^2^ ^3^ ^8^ ^13^ ^14^ ^18^ ^19^ Since 2008, the Oxford laboratory has reported NMDAR-Abs as Low Positive or Positive. Here, in order to inform the evaluation of future patients, we analysed the clinical features, antibody levels and treatment responses of the patients referred for testing from two specialist centres. We first divided the patients’ conditions into four categories of Definite paraneoplastic, Definite non-paraneoplastic, Possible and Unlikely autoimmune disease based on the clinical features and any responses to immunotherapies. Although the two Definite categories (n=25, all but 2 successfully treated with immunotherapy) contained the highest proportion (18/25, 72%) of Positive NMDAR-Abs, these were also seen in a proportion (5/18, 28%) of those in the Possible category but only in 2/13 (15%) of the Unlikely category. Conversely, Low Positive NMDAR-Abs were identified in seven of the Definite non-paraneoplastic cases and in several cases, both Definite and Possible, rose to Positive levels during the disease course. Ignoring results of sera scoring 1, as we did from 2011, would have reduced the number of Unlikely and Possible patients, including two with tumours, but also two of those designated as Definite.

We found that by using live cell assays, which would seem the most appropriate to detect the extracellular binding of antibodies that are potentially pathogenic,^5^ ^6^ serum levels were always higher than CSF levels and the latter were not always Positive in those with lower serum levels, regardless of whether they were assigned as Definite or Possible NMDAR-Ab encephalitis. Although the number of serum:CSF pairs was low, and often not taken at onset, a review of results on over 800 serum: CSF pairs over the same time period shows that 28 of 130 serum positive pairs were CSF negative (AV unpublished observations).

A recent report of assays in patients with ‘new onset neuropsychiatric symptoms and positive NMDAR-Abs’ demonstrated that live cell assays using serum were less sensitive than those with CSF on fixed-cell assays.^20^ However, since the patient definition relied on the positive CSF NMDAR-Ab test and the characteristics of the serum negative patients were not given, it is not possible to compare with our data. Clearly, a comparison of our approach with the fixed cells and tissue techniques used by others^20^ ^21^ would be valuable, but our study was retrospective up to the end of 2012 and the majority of the samples are no longer available. Equally important will be to compare both methods with the commercially available tests in order to inform better the clinical laboratories that are performing them. A workshop to agree the core features required, allowing some spreading of the phenotypes encountered, and a multicentre distribution of anonymised sera should be established for that purpose.

Some of the patients had unusual presentations or disease associations. NMDAR-Abs were identified in neurodegenerative disease, particularly in three cases of Alzheimer’s disease (one labelled Definite NMDAR-Ab encephalitis due to postmortem evidence of encephalitis and initial response to treatment) and two cases of motor neuron disease. These cases had atypical phenotypes at presentation, which prompted antibody testing, and therefore do not represent cases of typical Alzheimer’s disease or motor neuron disease encountered in the clinic. The prevalence of NMDAR-Ab in unselected Alzheimer’s disease and motor neuron disease is not known and would be of interest for future study. It is most likely that the antibodies are epiphenomena in these cases and not pathogenic, but a contribution to clinical features might be predicted given the pathogenicity of these antibodies. The case of leukodystrophy with NMDAR-Abs, and small vessel cerebrovascular disease is intriguing since white matter lesions resembling demyelination have been seen recently in paediatric^18^ and adult patients with NMDAR-Abs,^19^ and NMDARs are expressed in oligodendrocytes.^22^ The case of Definite NMDAR encephalitis after anti-TNF therapy for ankylosing spondylitis, a therapy usually associated with CNS demyelinating disease,^23^ could be related, although this patient had no evidence of demyelination on MRI.

Twelve (28.6%) of 42 cases in whom a search for tumour was carried out had tumours, including two in those with Low Positive antibodies (score 1). The presence of NMDAR autoantibodies should always prompt a consideration of screening for malignancy,^24^ but whether such low levels of autoantibodies justify a tumour search is not yet clear. The follow-up in our retrospective study was long, though it does remain possible that tumours may emerge years after the presentation in a small proportion.

We hoped that there might be some distinguishing features that helped to define a subgroup of Low Positive or Positive Possible patients who might respond to immunotherapies. There were no specific clinical or paraclinical features that were distinctive, but from the data presented (figure 2D) those with three or more of the core clinical features, including an abnormal CSF, could be considered for immunotherapies irrespective of whether their NMDAR-Abs are Positive or Low Positive.

The study was a retrospective series with some censored data. Since patients often presented first to distant referral centres before transfer to Addenbrooke’s Hospital or the National Hospital for Neurology and Neurosurgery, the clinical phenotype at presentation was based on archived clinic letters. As might be expected, the clinicians defined as Definite those cases with many of the core features, and as Possible mainly those cases with fewer features where they chose not to give immunotherapies. Although relating the number of clinical features to these definitions therefore involves some circular arguments, this does not apply to the antibody levels. In many cases, the treating clinicians made a clinical judgement prior to the knowledge of the antibody status, or did not treat the patients with immunotherapies if they did not think the antibody result was relevant. This indicates that Positive or Low Positive results did not necessarily mislead the clinician, but an opportunity for treatment may have been missed in some of the Possible patients who had three or more core features. Whether the use of fixed permeabilised cell-based assays and immunohistochemistry, as recently recommended,^20^ would have proved equally sensitive and more specific for the Definite patients could not be assessed in this retrospective analysis.

These data also demonstrate some of the difficulties encountered in routine clinical practice with respect to the reporting of results that will be helpful to the treating clinician. Low serum levels of NMDAR-Abs, often associated with a negative CSF, may rise subsequently, and appear to be relevant in a proportion of cases. Conversely, in other patients, Low Positive NMDAR-Abs may reflect the presence of a neurodegenerative or other disorder, unlikely to be immunotherapy responsive. Regardless of the antibody detection, the careful clinical assessment of suspected NMDAR-Ab-mediated disorders remains critical to management.

## Supplementary Material

Supplementary data

## Figures and Tables

**Table 1:** Clinical features of the cases according to likelihood categories and NMDAR-Ab levels

Likelihood category	Definite (n=25)			Possible (n=18)		Unlikely (n=13)	
	Definite paraneoplastic	Definite non-paraneoplastic					
NMDAR-Ab Total number	Positive N=9	Positive N=9	Low Positive N=7	Positive N=5	Low Positive N=13	Positive N=2	Low Positive N=11
Age range (median)	17–48 (25)	16–41 (26)	18–68 (44)	22–80 (65)	22–71 (54)	72, 77	17–67 (49)
F:M	8:1	7:2	3:4	3:2	4:9	0:2	4:7
CSF NMDAR-Ab positive any time	3/3	1/1	1/4	0/1	0/2	0/1	0/0
Psychiatric	6/8	8	3	3	4/12	0	3
Encephalopathy	8/8	9	6	5	6	0	0
Cognitive	8/8	9	7	5	6/12	2	4/10
Seizures	7/8	7	4	2	5	1	3
Extrapyramidal	6/8	7	5	2	3/12	0	2
CSF lymphocytosis or OCBs*	5/7	8	4/6	4/4	4/11	1/2	1/10
3 or more core clinical features (including abnormal CSF)	8/8	9	7	5	6	0	1
Investigations							
MRI MTL	1	0	1	1	2	0	0/10
MRI WM	0	2	3	0	3	1	1/10
MRI atrophy	0	1	1	3	3	0	1/10
EEG epileptic	2	3/8	1/2	1/3	2/10	ND	2/8
EEG slowing	8	8/8	2/2	2/3	5/10	ND	2/8
Outcomes							
Treated	9	9	7	2	4	2	0
Responded	8	8	7	0	1	0	0

* In many cases, OCB were not systematically looked for.

Phenotypes are given from the retrospective clinical data where available. Denominators are given in cases of censored data. Initially normal but subsequently abnormal tests around the time of presentation (MRI, EEG, CSF) were counted as abnormal in this analysis, unless stated in the online supplementary table S1. CSF, cerebrospinal fluid; F:M, female:male; MTL, medial temporal lobe abnormalities; ND, not done; NMDAR-Ab, *N*-methyl-d-aspartate receptor autoantibody; OCB, oligoclonal band; WM, white matter abnormalities.
