# Supplementary material for: Clinical relevance of serum antibodies to extracellular N-methyl-d-aspartate receptor epitopes
Source: J Neurol Neurosurg Psychiatry. Author manuscript; Available in PMC 2018 Jul 23. (PMC6055984; doi:10.1136/jnnp-2014-308736)
Supplement: Supplementary data [file NIHMS78483-supplement-Supplementary_data.pdf]

Supplementary Table 1. Clinical features of 56 NMDAR antibody-positive patients

Case number	**Age, sex**	***NMDAR-Ab CBA scores*** (Paired serum (1:20), CSF (1:1), days since first sample) (approximate length of history prior to antibody testing, months (m)	**Clinical syndrome**	**Investigations**	**Final clinical diagnosis**	**Response to treatment**	**Primary autoimmune likelihood (follow up, months (m))**
Definite paraneoplastic (listed by NMDAR-Ab first sample)							
1	17 F	*4, 4* (na)	Protracted encephalitis requiring intensive care unit stay (censored data).	CSF unknown; EEG slowing, rhythmic delta and occasional spikes; MRI normal.	NMDAR-Ab encephalitis; teratoma (found on oophorectomy)	No; (steroids, PEX, IVIG) (died of bowel sepsis)	Definite paraneoplastic (14m)
2	21 F	*4, (3.5, CSF1.5, 6m), 4* (na)	Headaches, hallucinations, confusional state, movement disorder, dysautonomia, seizures, dyskinesias.	CSF unknown; EEG encephalopathic; MRI normal	NMDAR-Ab encephalitis; ovarian teratoma; post mortem CA4 encephalitic changes	Yes (tumour removal, IVIG, steroids, rituximab).	Definite paraneoplastic (9m) died
3<br><br>Included in Irani et al 2010	32 F	*4, 4, 1.5* (0m)	Psychosis, seizures, coma, rhythmic movements	CSF normal; EEG slowing; MRI normal	NMDAR-Ab encephalitis; ovarian teratoma	Yes (steroids, PEX, tumour removal)	Definite paraneoplastic (55m)

4	25 F	*4, 4* (1m)	Behavioural change, seizures, coma, movement disorder	CSF normal; EEG episodic anterior slow discharges; MRI normal	NMDAR-Ab encephalitis; ovarian teratoma	Yes (IVIG, PEX, tumour removal, steroids, rituximab)	Definite paraneoplastic (3m)
5<br><br>Case 1 in Davies et al. 2010[17]	20 F	*4, 2.5 (na)*	Behaviour change, seizures, encephalopathy, dyskinesias. Intubated for several months. Previous (contralateral) teratoma removed 24m earlier.	CSF 74 Lymphs, 6 PMNs; OCB; EEG epileptiform then encephalopathic; MRI normal	NMDAR-Ab encephalitis; previous and new ovarian teratoma	Yes (steroids, tumour removal, PEX).	Definite paraneoplastic (10m)
6	29 F	*4* (na)	Psychosis, encephalopathy, seizures.	CSF 57 WCC; EEG slowing; MRI normal	NMDAR-Ab encephalitis; ovarian teratoma	Yes (tumour removal, PEX)	Definite paraneoplastic (27m)
7<br><br>Case 6 in Davies et al. 2010[17]	22 F	*3, 3* (na)	Forgetfulness then headaches, fever and confusional state, seizures and coma, dysautonomia, dystonia. Positive ANA and anti-Ro antibodies, anti-dsDNA negative. Reactivation of TB on therapy, protracted course.	CSF 8 lymphs; OCB; EEG encephalopathic; MRI normal	NMDAR-Ab encephalitis; bilateral ovarian teratomas	Yes (teratoma removal, steroids, IVIG, cyclophosphamide, rituximab)	Definite paraneoplastic (56m)
8	25 F	*(3, CSF 1, 40d), 4, 1.5* (na)	Encephalitis with initial psychosis, catatonia, dystonia then coma.	CSF 21 lymphs; EEG slow triphasic waves, encephalopathic; MRI normal	NMDAR-Ab encephalitis; ovarian teratoma and papillary thyroid cancer	Yes (IVIG, PEX, steroids, removal of tumours)	Definite paraneoplastic (24m)
9<br><br>Zandi et al 2009[16],Irani et al[2]	48 M	*2, (2, CSF1, not cleara)* (0m)	Amnestic syndrome, seizures, relapsed Hodgkin lymphoma (‘Ophelia syndrome’)	CSF 4 WCCs; then 10 lymphs; EEG normal; MRI medial temporal lobe high signal	Hodgkin lymphoma associated limbic encephalitis (Ophelia	Yes (PEX)	Definite paraneoplastic (6m) but died later

					syndrome)		
Definite (non-paraneoplastic, listed by NMDAR-Ab first sample)							
10	21 F	*4, 3* (na)	Psychosis, apraxia, behavioural change, seizures	CSF 29 WCC, OCB; EEG encephalopathic, MRI normal then meningeal lesions	NMDAR-Ab encephalitis; no tumour found	Yes (steroids, plasmapheresis, IVIG, AZA)	Definite (35m)
11	23 F	*4, 4* (na)	Psychosis, seizures, dystonia, autonomic dysfunction. No follow up.	CSF OCB, EEG slowing, MRI normal	NMDAR-Ab encephalitis; no tumour found	Yes (PEX, steroids)	Definite (2m)
12	40 F	*4, 0* (na)	Behavioural disturbance, musical hallucinations, seizure, encephalopathy, movement disorder. Cardiac asystole.	CSF 13 WCC, OCB; EEG slowing, MRI few areas white matter high signal	NMDAR-Ab encephalitis; no tumour found	Yes (corticosteroids, IVIG)	Definite (40m)
13<br><br>Case 2 in Davies et al. 2010[17]	41 M	*4,3* (1m)	Behaviour change, hallucinations, seizures, coma, dysautonomia, rhythmic orofacial and limb movements, hyperthermia	CSF normal but then 40 WCC (95% lymph), raised protein and OCB.; EEG epileptiform then encephalopathic; MRI normal	NMDAR-Ab encephalitis; no tumour found	No (Steroids, IVIG x 2, plasmapheresis x 2)	Definite (6m) died
14<br><br>Case 3 in Davies et al. 2010[17]	29 M	*3, 1.5*	Behaviour change and seizures, auditory hallucinations, delusions, coma and dyskinesias. Prolonged rehabilitation, now in sheltered housing.	CSF 12 WCC; transient positive HSV1, OCB. EEG slow, MRI normal	NMDAR-Ab encephalitis; no tumour found	Yes, (steroids, plasmapheresis, rituximab)	Definite (49m)

15	22 F	*(2, CSF1, 0d), 2.5, 1.5* (na)	Behaviour change, amnesia, movement disorder. Relapses late 2012.	CSF 20 WCC; EEG focal temporal and occipital changes; MRI normal	NMDAR-Ab encephalitis; no tumour found	Yes (IVIG, PEX, rituximab, AZA)	Definite (uk)
16	16 F	*2.5, 3, 1* (2m)	Musical hallucinations, seizures, cerebellar syndrome (subsequent relapsing cognitive and psychiatric symptoms)	CSF normal; EEG high amplitude slow waves and spikes, MRI normal	NMDAR-Ab encephalitis; no tumour found	Yes (steroids, PEX, IVIG, rituximab)	Definite (58m)
17<br><br>Case 5 in Davies et al. 2010[17]	26 F	*2* (na)	Headache, acute confusion, memory loss and word finding difficulties, athetoid then rhythmic movements.	CSF 114 WCC; EEG encephalopathic; MRI normal	NMDAR-Ab encephalitis; no tumour search.	Yes (Steroids, IVIG)	Definite (24m)
18<br><br>Case 4 in Davies et al. 2010[17]	28 F	*2* (na)	Headache, fever, agitation, hallucinations, seizures, dyskinesias.	CSF 60 lymphs; OCB; EEG encephalopathic, one seizure; MRI normal then WM lesions	NMDAR-Ab encephalitis; no tumour search	Yes (steroids)	Definite (48m)
19*	26 F	*1.5, (2.5, CSF1.5, 25d), 4, 2.5* (~1 m).	Behaviour change, hallucinations, movement disorder	CSF Lymphocytic; EEG encephalopathic; MRI possible frontal ischaemia	NMDAR-Ab encephalitis; no tumour found at oophorectomy. Remains dependent in nursing residential care.	Yes (steroids, IVIG, PEX, rituximab, AZA)	Definite (18m)
20*	33 F	*1.5, (1, CSF0, 40d), 2.5* (na)	Headache, seizures, coma, dysautonomia	CSF not known; EEG sharp waves; MRI normal	NMDAR-Ab encephalitis; no tumour found	Yes (steroids, IVIG, rituximab)	Definite (uk)
21	68 M	*1.5, 1* (14m)	Seizures, amnesia, cerebellar and bradykinesia, coeliac	CSF normal, EEG borderline theta;MRI atrophy and	Post mortem Alzheimer's Disease type	Yes (PEX, steroids), then deteriorates and	Definite (20m) then

			disease, subsequent dementia	periventricular WM high signal	pathology and basal ganglia and hippocampal encephalitic changes; no tumour found	dies	died
22*	46 M	*(1.5, CSF0, 0d), (0, CSF0)* (8m)	Complex partial seizures, amnesia. Ankylosing spondylitis and previous anti-TNF therapy (adalimumab)	CSF normal, EEG left temporal slowing, MRI high signal left temporal lobe	NMDAR-Ab encephalitis (post anti-TNF); no tumour found	Yes (steroids, PEX)	Definite (40m)
23	57 F	*1.5* (1m)	Confusion and delusions, psychosis, catatonia, orofacial dyskinesia	CSF 225 lymph; OCB; EEG no epileptic activity; MRI, few areas of WM high signal	NMDAR-Ab encephalitis; no tumour found	Yes (PEX, steroids MMF)	Definite (11m)
24	18 M	*1, 0, CSF0* (1m)	Psychosis, insomnia, headache, agitation, then sustained remission	CSF 22 WCC; EEG, MRI normal	NMDAR-Ab psychosis; no tumour found	Yes (IVMP)	Definite (9m)
25	44 M	*1, (0, CSF0 5m), 0* (11m)	Cerebellar syndrome, with asymmetric myoclonus, dyspraxia and bradykinesia of right arm, memory problems	CSF raised protein, OCBs; EEG, MRI normal	NMDAR-Ab encephalitis; no tumour found	Yes (PEX)	Definite (7m)
Possible (paraneoplastic; non-paraneoplastic, listed by NMDAR-Ab first sample)							
26	65 F	*1*(2m)	Seizures, head jerks, cognitive impairment, rheumatoid arthritis, non-small cell lung adenocarcinoma subsequently found	CSF normal; EEG slow; MRI normal	Localisation related seizures; non-small cell lung cancer	Not tried (possible spontaneous resolution of symptoms)	Possible paraneoplastic (22m) died two m later
27	79 M	*1* (2m)	Weight loss, encephalopathy, agitation and unsteadiness.	CSF 34 lymphs; EEG frontal slowing; MRI	No diagnosis made in life;	Not tried	Possible paraneoplastic

				non-specific WM changes	oesophagus mass; prostate adeno-carcinoma		(0m) died
28	22 F	*4, 4, 4* (na)	Headache, fatigue, hypersomnolence, fever, coma. Initial working diagnosis of neuro-Behçet's disease.	CSF 30 WCC; EEG slow with some sharp features; MRI normal initially except transverse and sinus thrombosis. Later MTL inflammation	NMDAR-Ab encephalitis and venous sinus thrombosis; no tumour found	No (Steroids, infliximab, methotrexate; plasma exchange and rituximab following NMDAR antibody result).	Possible (7m)
29	80 F	*4, 4, 1.5* (3m).	Behavioural change and facial dyskinesia (3m). Two years of depression, received electroconvulsive therapy.	CSF and EEG nd; MRI normal	Depression; no tumour search	No (steroids)	Possible (0m)
30	52 F	*4, 2.5, 1.5* (12m)	Encephalitis, seizures, myeloradiculopathy. Chronic seizures and cognitive impairment.	CSF 34 WCC, raised protein, OCB, ACE raised; EEG slowing; MRI signal change T6 to conus, cerebellar haemorrhage	Biopsy proven neurosarcoidosis with systemic sarcoidosis; no tumour found	Not tried (already on steroids and methotrexate for sarcoid)	Possible (no follow-up)
31	33 M	*2.5*(na)	Relapsing encephalitis in youth. Mild fluctuating psychiatric symptoms since then. Antibodies tested when well.	CSF 12 WCC in 1996 (counted in this analysis); EEG nd; MRI normal	Idiopathic encephalitis and psychiatric syndrome; no tumour search	Not tried (relevance unclear, mild symptoms)	Possible (44m)
32	67 M	*2, (1.5, CSF0 22m), 2.5* (0m)	Porencephalic cyst, longstanding schizophrenia. Episode of delirium. (0m) (Relapse with seizures 1 year later, and further seizures and	CSF, EEG and MRI nd at presentation; EEG discharges at first relapse and slow at second, CSF cellular 1st relapse, with	Localisation related seizures and chronic schizophrenia; no tumour	Not tried (spontaneous resolution)	Possible (28m)

			possible stroke at 22 months)	MRI high signal right hemisphere at 22 months.	found.		
33*	22 M	*1.5* (0m)	Acute psychosis: visual and auditory hallucinations with cannabis (0m) Childhood generalised seizures.	CSF, EEG nd;MRI normal	First episode psychosis; no tumour search	Not tried; (lost to follow up)	Possible (no follow-up)
34	26 F	*1.5*, *2.0*, *1.5* (2m)	Relapsing psychosis	CSF and EEG normal; MRI subtle increased signal cingulate, insular and hippocampi	First episode psychosis; no tumour found	Yes (steroids then olanzapine)	Possible (13m)
35	39 M	1.5, 2.5, 1 (14m)	Paroxysmal hyperkinetic movement disorder, cognitive dysfunction and subsequent psychiatric symptoms	CSF normal; EEG intermittent slowing; MRI normal	No clear diagnosis made; no tumour found	No; (steroids only)	Possible (no response but only given steroids; no follow-up)
36	68 M	*1.5*, *1* (0m)	Spontaneously rapidly resolving acute encephalitis with confusion and poor communication post respiratory infection	CSF 120 PMNs, 20 lymphs, raised protein;EEG slowing;MRI probable small vessel disease	Possible NMDAR-Ab encephalitis; no tumour found	Not tried (spontaneously recovers)	Possible (6m)
37*	56 F	*1.5* (1m)	Acute mania	CSF nd; EEG and MRI normal	Manic episode; no tumour search	Not tried; (lost to follow up)	Possible (no follow-up)
38*	54 M	*1.5*, *2*, *1* (60m)	5 years progressive difficulty using right arm, and gait disturbance. Myoclonus and pyramidal signs. Stimulus sensitive jerky tremor. Polycythaemia.	CSF normal tau and abeta; EEG nd; MRI atrophy and small vessel disease	Possible corticobasal degeneration; no tumour search	Not tried (not felt to be relevant at the time)	Possible (0m)
39	33 M	*1*, *2.5* (84m)	Refractory localisation related epilepsy. Encephalitis	CSF 38 WCC; EEG normal; MRI	Hippocampal sclerosis; no	Not tried	Possible

			considered in admission 6 years prior to this presentation (fever, amnesia)	hippocampal sclerosis	tumour found		(27m)
40	56 M	*1, 1.5, (0.5, CSF0 2.3y)* (144m)	Recurrent partial seizures and epilepsia partialis continua, and increasing frequency of seizures over 12 years. Tremor.	CSF raised protein; EEG L hemispheric slowing; MRI progressive hemiatrophy	Rasmussen's encephalitis; no tumour found	No (steroids, PEX, AZA)	Possible (46m)
41	48 M	*1, 0* (0m)	Post respiratory tract infection relapsing encephalitis (fluctuating drowsiness, spontaneous resolution). (0m) Antibodies not measured in first episode 6 years prior to this episode.	CSF 20 WCC, raised protein; EEG nd; MRI normal	Idiopathic encephalitis; no tumour found	Not tried (spontaneous resolution)	Possible (9m)
42	62 M	*1* (0m)	Abdominal pain then seizures, hyponatraemia. (0m) Epilepsy for 4 years, recent bowel surgery for pseudomembranous colitis (5 m prior)	CSF normal; EEG asymmetrical with sharp features; MRI atrophy	No diagnosis made in life; no tumour search	Not tried; (result not known in life)	Possible (0) died
43	71 F	*1* (3m)	Seizures requiring sedation (3m)	CSF normal;EEG epileptiform;MRI high signal MTL, left parietal lobe haemorrhage	Diagnosis not made in life; no tumour found	No (steroids)	Possible (4m) died
Unlikely (remaining cases, listed by tumour then NMDAR-Ab first sample)							
44*	77 M	*1.5*	Cerebellar syndrome (6m)	CSF and EEG nd; MRI posterior fossa mass	Glioma; died; no autopsy	Not tried; DEX only	Unlikely (2m) died
45	72 M	*(2.5, CSF0, 0)* (30m)	2.5 years of amnesia suggestive of mild Alzheimer's disease, isolated generalised seizure, few	Small IgG kappa 12g/L paraprotein. CSF slightly raised protein (scored normal); EEG nd; MRI	Alzheimer's disease; no tumour found	Not tried	Unlikely (26m)

			episodes of deja vu. Deep vein thrombosis	normal	found		
46	28 M	*2*	Probable relapsing cerebral vasculitis, first episode with parenchymal haemorrhage 6 years prior, progressive infarcts and narrowing medium sized vessels on angiography despite immunosuppression	CSF 146 lymph; EEG n.d., MRI WM ischaemia.	CNS vasculitis; no tumour found	n/a (partial effect - cyclophosphamide, steroids, azathioprine)	Unlikely (31m)
47	17 M	*1.5*, *0* (60m)	Subacute ataxia and jerky nystagmus; known maple syrup urine disease (MSUD) without biochemical evidence of decompensation (1m)	CSF, EEG, MRI normal	Probable exacerbation of maple syrup urine disease; no tumour search	Not tried (spontaneously improves)	Unlikely (13m)
48	63 M	*1.5* (40m)	Focal seizures and migraine	CSF normal; EEG L epileptiform focus; MRI normal	Cryptogenic epilepsy; no tumour found	Not tried	Unlikely (0m)
49	67 M	*1*, *3*, *1.5* (48m)	Cognitive dysfunction and complex partial epilepsy	CSF normal; EEG intermittent slowing L temporal; MRI mild atrophy (scored normal in analysis)	Possible early Alzheimer’s Disease; no tumour found	Not tried	Unlikely (37m)
50	33 M	*1*, *1*, *0* (10m)	Motor neuron disease. Upper limb weakness and cramps, initially thought to be multifocal motor neuropathy	CSF OCBs; EEG and MRI nd	Motor neurone disease; no tumour found	No (IVIG)	Unlikely (51m)
51	45 F	*1*, *0*, *0* (60m)	Migraine, small meningioma, malaise, anti-purkinje antibody weak positive, Raynaud’s phenomenon,	CSF, EEG and MRI normal	No clear diagnosis made; no	Not tried	Unlikely (23m)

			bipolar disease, previous possible temporal lobe epilepsy. Subsequent loss of antibody		tumour found		
52	49 F	*1*(na)	Depression, mutism, previous possible systemic lupus erythematosus and polymyositis.	CSF and EEG normal; MRI non-specific WM signal (normal in analysis)	Depression	n/a (AZA)	Unlikely (0m)
53	49 F	*1* (13m).	Progressive cerebellar syndrome	CSF and EEG normal; MRI cerebellar, peduncle and pontine volume loss	Idiopathic late onset cerebellar ataxia; no tumour found	Not tried	Unlikely (18m)
54	52 M	*1* (na)	3 years of a frontal dementing illness, self-neglect, disinhibition. Father had a late onset Parkinsonian syndrome with similar brain imaging findings	CSF normal; EEG some slowing; MRI confluent WM change	Probable leukodystrophy, genetics undetermined; no tumour search	Not tried	Unlikely (1m)
55	55 F	*1*, *0* (30m)	Stiffness, difficulty walking, startle, pyramidal signs, eventual bulbar involvement. (30m)	CSF normal; EEG unknown; MRI normal	Upper motor neuron predominant motor neurone disease	No (PEX)	Unlikely (18m)
56	57 M	*1* (120m)	Recurrent focal seizures over 10 years, previous aneurysmal subarachnoid haemorrhage 43 years prior.	CSF normal; EEG L frontotemporal discharges; CT L temporal lobe volume loss with aneurysm clips.	Refractory epilepsy	Not tried	Unlikely (48m)

Cases #27, 42, and 43 died before the information about the antibody was available and their likelihood was assigned by the authors.

Abbreviations: AZA azathioprine; ACE angiotensin converting enzyme; CSF cerebrospinal fluid; DEX dexamethasone; EEG electroencephalogram; HSV1 herpes simplex virus 1; IVIG intravenous immunoglobulin; L left; lymphs lymphocytes; MRI magnetic resonance imaging; MSUD maple syrup urine disease; na not available; nd not done; OCB oligoclonal bands seen in CSF; PEX plasmapheresis; PMN

polymorphonuclear leukocytes; uk unknown; WCC white cell count; WM white matter. *The NMDAR-Ab scores at first sampling (1.5) were reported as positive in seven patients, before this value was re-designated as Low Positive. In cases with more than three longitudinal scores the 1st serum CBA score and last CBA score are given, with the maximum CBA score given if not one of the former, and a maximum of one CSF paired score is given if available.
